# The Effect of Fenugreek (*Trigonella foenum‐graecum*) Supplementation on Liver Function Enzymes; a Systematic Review and Meta‐Analysis of Controlled Clinical Trials

**DOI:** 10.1002/hsr2.72960

**Published:** 2026-08-04

**Authors:** Mohsen Mohit, Melika Mahmoudizadeh, Mohammad Hashem Hashempur, Mitra Soltani, Asma Kazemi

**Affiliations:** ^1^ Student Research Committee, Endocrinology and Metabolism Research Center Shiraz University of Medical Sciences Shiraz Iran; ^2^ Canadian College of Integrative Medicine (CCIM) Montreal Quebec Canada; ^3^ Research Center for Traditional Medicine and History of Medicine, Department of Persian Medicine, School of Medicine Shiraz University of Medical Sciences Shiraz Iran; ^4^ Department of Clinical Nutrition, School of Nutritional Sciences and Dietetics Tehran University of Medical Sciences Tehran Iran; ^5^ Nutrition Research Center Shiraz University of Medical Sciences Shiraz Iran

**Keywords:** fenugreek, functional foods, hepatoprotective, integrative medicine, liver diseases, medicinal plants, transferases, *Trigonella foenum‐graecum*

## Abstract

**Background and Aim:**

The present systematic review and meta‐analysis aimed to investigate the effect of fenugreek supplementation on key liver enzymes, including alkaline phosphatase (ALP), alanine aminotransferase (ALT) and aspartate aminotransferase (AST).

**Method:**

PubMed, Web of Science, and Scopus databases were searched from 1990 up to April 2025. Clinical trials examining the effects of fenugreek supplements on liver enzymes, specifically ALP, ALT and AST, were included in the study if they met the inclusion criteria. Random‐effects meta‐analysis was employed to calculate pooled weighted mean differences (WMD) and standardized mean differences (SMD) with 95% confidence intervals (95% CI). To evaluate statistical heterogeneity, the I‐squared (I^2^) statistic was used.

**Results:**

Sixteen studies were included, involving a total of 1171 participants. Results indicated that fenugreek supplementation had no significant effect on ALP (SMD = −0.21, 95% CI = −0.44, 0.03), AST (WMD = 0.03 IU/L, 95% CI = –0.12 to 0.18), and ALT (WMD = −1.41 IU/L, 95% CI = −3.07, 0.26). However, a significant reduction in ALP was observed for a dose of more than 1 g/day.

**Conclusion:**

Fenugreek didn't affect ALT and AST. However, there is potential benefit at doses ≥ 1 g/day regarding ALP. Further research should investigate effective doses in homogeneous populations and evaluating safety.

**Abbreviations**
ALP: alkaline phosphatase
ALT: alanine aminotransferase
AST: aspartate aminotransferase
BMI: body mass index
CI: confidence interval
NAFLD: non‐alcoholic fatty liver disease
PRISMA: Preferred Reporting Items for Systematic Reviews and Meta‐Analyses
SD: standard deviation
SMD: standardized mean difference
T2DM: type 2 diabetes
WMD: weighted mean differences

## 1 Introduction

Two million people pass away annually from hepatic disorders. Liver diseases are responsible for approximately every 1 out of 25 deaths each year whereas cirrhosis and hepato‐carcinoma are among the leading contributors to liver‐related mortality [1]. Non‐alcoholic fatty liver disease (NAFLD) is the most prevalent liver disease worldwide, affecting almost one‐third of the global population. It can eventually lead to cirrhosis and hepatocellular carcinoma [2]. In line with this globa lburden, NAFLD affects almost 33% of the Iranian population and 39.43% of Middle East population [3, 4]. Regarding the increasing incidence of metabolic diseases mainly diabetes mellitus (T2DM), socio‐economic factors, following unhealthy dietary patterns, and aging, it's anticipated that the prevalence and burden of liver disease will escalate rapidly [1, 2].

Nowadays, there is increasing interest in functional foods for managing various health conditions. Patients with chronic diseases and metabolic disorders are among the most prevalent users of these options [5, 6, 7, 8, 9]. Fenugreek, scientifically known as *Trigonella foenum‐graecum*, is widely utilized as a spice and medicinal herb [10]. However Iran and India are major producing countries, due to the adaptation of the seed to different growth conditions, it is extensively cultivated in the world [11]. As a recognized functional food, it is a rich source dietary laxative fibers, carbohydrates, proteins, lipids, steroidal sapogenins, trigonelline, saponins, polyphenols, and various bioactive ingredients, in addition to vitamins and minerals. Consequently, this plant is extensively employed in medical conditions owing to its therapeutic properties including anti‐diabetic, anti‐lipidemic, anti‐inflammatory, antioxidative, anti‐cancer, hypotensive, cardioprotective, antimicrobial, anti‐obesity, analgesic and galactogogue effects [12, 13, 14, 15, 16, 17]. Furthermore, fenugreek is recognized as an important medicinal botanical across multiple traditional medical systems, such as Ayurveda and traditional Persian medicine. It is known as *Shanbalileh* in Persian. Fenugreek has been utilized in the management of various medical conditions, including but not limited to respiratory ailments, gastrointestinal diseases, gynecological disorders, and dermatological conditions [15, 18, 19, 20, 21].

It is also assumed that the fenugreek plant has hepatoprotective effects. For instance, several studies have demonstrated that fenugreek can attenuate drug‐ and chemical‐induced hepatotoxicity, probably through anti‐inflammatory and anti‐oxidative properties [22, 23, 24]. In addition, the favorable effects of fenugreek on fatty liver have been investigated. It has been shown that fenugreek prevents fat deposition in the liver mainly through hypoglycemic and anti‐inflammatory mechanisms [25, 26, 27]. Conversely, limited case‐report studies have suggested that fenugreek may lead to liver damage [28, 29]. Although the majority of experimental studies have shown the beneficial effects of fenugreek on liver health, there is no comprehensive consensus on the implication of fenugreek in liver disorders. While some studies have demonstrated that fenugreek can improve hepatic status mostly evaluated by the levels of aspartate aminotransferase (AST), alanine aminotransferase (ALT), and alkaline phosphatase (ALP) [30, 31, 32]; some failed to find such significant effects [33, 34, 35, 36]. Considering inconsistent evidence from previous studies, this meta‐analysis was conducted to investigate the effect of fenugreek on liver status evaluated by liver function enzymes including AST, ALT, and ALP.

## 2 Methods

The design and implementation of the current meta‐analysis were conducted with the guideline outlined in the Cochrane Handbook for Systematic Reviews [37]. Additionally, the reporting of this meta‐analysis adhered to the Preferred Reporting Items for Systematic Reviews and Meta‐Analyses (PRISMA) statement [38] was used in reporting of the this meta‐analysis. The study's protocol was registered in the International Prospective Register of Systematic Reviews (PROSPERO) database under registry code CRD42024626893.

### 2.1 Search Strategy

The electronic databases, including PubMed/Medline, Scopus, and Web of Science were searched from 1990 to April 2025, using the following terms and word combinations in the studies’ titles or abstracts: (Trigonella OR Fenugreek OR Fenugreeks OR Foenumgraecum OR foenum OR graecum) AND (Intervention OR “controlled trial” OR randomized OR random OR randomly OR randomi* OR placebo OR “clinical trial” OR Trial OR “randomized clinical trial” OR RCT OR trial OR “Cross‐Over Studies” OR “Cross‐Over” OR “CrossOver Study” OR parallel OR “parallel study” OR “parallel trial” OR blind). Since liver enzyme levels are often reported as secondary outcomes in various studies, we conducted a broader search that included both fenugreek and clinical trials to ensure that no relevant research was overlooked by limiting our search to specific outcome keywords.

### 2.2 Study Selection

The eligibility of randomized controlled trials (RCTs) was evaluated based on the PICO framework, which included the following criteria: Participants: Human adults; Intervention: Fenugreek supplementation; Comparator: Placebo; Outcomes: liver function enzymes (ALP, ALT, AST, and GGT). Two independent investigators (M.M and M.M.Z) screened the articles initially by titles and abstracts, followed by a full‐text review. Any disagreement between the investigators were resolved through discussion with the research team. Studies were included in this meta‐analysis if they met the following eligibility criteria: a) parallel or cross‐over randomized control trials (RCTs); b) involved human adults (aged > 18 years); c) administered fenugreek seed in any form orally; d) evaluated the effects of fenugreek's effects on at least one liver function enzymes (ALP, ALT, AST); and e) reporting pre‐ and post‐intervention values or net changes in outcome parameters for each group.

### 2.3 Data Extraction

Two investigators (M.M and M.M.Z) extracted respective data from each included study, which encompassed: a) study characteristics (first author, year of publication, country of origin, study design, and overall sample size); b) participants’ information (gender, mean age, mean BMI, and health status); c) data of the intervention and comparison (type and dosage of administration in fenugreek and control groups, and the duration of intervention); and d) Means and SDs of the outcome parameters at baseline and post‐intervention or their changes from baseline to the end of the intervention.

### 2.4 Quality Assessment and Certainty of Evidence

The Cochrane Collaboration's risk of bias tool version 1 [39] was utilized to evaluate the quality of RCTs. Seven domains of methodological quality, including random sequence generation, allocation concealment, blinding of participants and personnel, blinding of outcome assessment, incomplete outcome data, selective reporting, and other sources of bias (chance bias and conflict of interest) were labeled as low, high, or unclear risk of bias for each study by two independent investigators. In cases of disagreement, a group discussion with other authors was held to reach consensus.

The certainty of evidence for each outcome was assessed using the GRADE framework, which rates evidence as high, moderate, low, or very low based on risk of bias, heterogeneity, indirectness, imprecision, and publication bias.

### 2.5 Statistical Analysis

All statistical analyses were performed using STATA version 14. The effect sizes for the outcomes of interest were expressed as weighted mean differences (WMD) for AST and ALT, and standardized mean differences (SMD) with 95% confidence intervals (CIs) for ALP. Since studies reporting ALP in different units or scales, SMD was used to standardize the results. The calculation of SD changes in trial groups was based on the formula proposed by Follmann et al. [40], with consideration of a correlation coefficient (R) = 0.5:SD = square root [(SD pre‐treatment)2 + (SD post‐treatment)2 – (2 R × SD pre‐treatment × SD post‐treatment)]. For studies that reported outcomes as median and interquartile range (IQR), the mean and standard deviation (SD) were estimated using established methods [41]. A random‐effects model was applied to account for potential variability across studies, as it provides a more conservative estimate in the presence of heterogeneity [42]. Heterogeneity among the included studies was assessed using the *I^2^* statistic. Sensitivity analyses were conducted to evaluate the robustness of the findings by sequentially excluding each study and assessing its impact on the overall effect size. Pre‐ specified subgroup analyses were performed to explore potential sources of heterogeneity, including dosage of fenugreek supplementation, duration of intervention, baseline liver enzyme levels, risk of bias, and participant characteristics (e.g., age, BMI, health status), and interaction testing was performed. Publication bias was assessed using visual inspection of funnel plots and Egger's regression test. A *p* < 0.05 was considered statistically significant for all analyses. For studies that reported outcomes as median and interquartile range (IQR), the mean and standard deviation (SD) were estimated using established methods [41]. All hypothesis tests were two‐sided, and a *p*‐value of less than 0.05 was considered statistically significant for all analyses.

## 3 Results

### 3.1 Search Results

**Figure 1:**
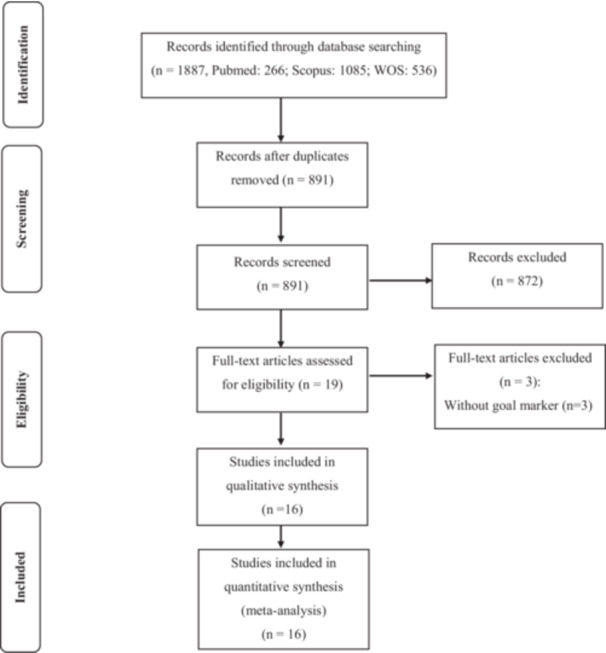
Flow diagram of the systematic review and meta‐analysis study selection process.

After conducting a comprehensive search in the Scopus, PubMed, and Web of Science databases, a total of 1887 documents were found, with 1085, 266, and 536 documents identified in each database, respectively. After removing duplicates, 891 documents remained. The titles and abstracts of the articles were reviewed to identify relevant studies. During this process, documents were excluded for various reasons, including irrelevance, animal studies, review articles, co‐supplementation, conference papers, letters to the editor, or sections of books. Ultimately, 16 articles were considered for analysis (Figure 1).

### 3.2 Study Characteristics

The characteristics of the included studies are summarized in Table 1. Half of studies involved participants with metabolic disorders conditions such as Type 2 Diabetes Mellitus/prediabetes (*n* = 6), NAFLD (*n* = 1), and polycystic ovary syndrome (n = 1). The mean age of participants ranged from 18 to 62.9 years. The sample sizes ranged from 23 to 208, with intervention durations varying from 6 to 12 weeks. The dosage of fenugreek varied, with seven of the studies administering 0.3 to 0.6 g, while others administering between 1 g to 15 g of fenugreek seeds powder or extracts daily. The majority of studies were conducted in India (*n* = 9), followed by Australia (*n* = 4), Iran (*n* = 2), and China (*n* = 1).

**Table 1:** Characteristics of eligible randomized controlled trials.

First author, year	Country	Study design	Participants (Intervention, Control)	Population	Mean age (Intervention, Control)	Type of administration	Duration of intervention	Dosage (g/d)
Lu, 2008	China	RCT, Parallel	69 [23, 43]	T2DM	54.43 (54.8, 53.7)	TFGs capsules	12 weeks	6.3
Wankhede, 2016	India	RCT, Parallel	55 [26, 29]	Healthy men	22.41 (23.21, 21.62)	Capsule (furostanol glycosides‐based fenugreek seeds extract (Fenu‐FG))	8 weeks	0.6
Inanmdar, 2016	India	RCT, Parallel	40 [20]	Women with primary dysmenorrhea	17.87 (17.7, 18.05)	Capsule of Fenugreek seed powder	12 weeks	6
Verma, 2016	India	RCT, Parallel	154 [44, 45]	T2DM	25‐60 years	Fenfuro® capsules	12 weeks	1
Rao, 2019	Australia	RCT, Parallel	84 [42]	Healthy men with benign prostate hyperplasia	62.9 (61.8, 64)	Testofen capsule	12 weeks	0.6
Deshpande, 2020	India	RCT, Parallel	23 [11, 12]	Human subjects with high‐fat mass	25.91 (27.42, 24.27)	Capsule of low molecular weight galactomannans‐based standardized fenugreek seeds extract (LMWGAL‐TF)	8 weeks	0.5
Hadi, 2020	Iran	RCT, Parallel	48 [24]	T2DM	47.55 (47.7. 47.4)	Fenugreek seed powder	8 weeks	15
Babaei, 2020	Iran	RCT, Parallel	24 [11, 13]	NAFLD	39.29 (39.08, 39.55)	Hydroalcoholic extract of Fenugreek seeds	3 months	1
Khanna, 2020	India	RCT, Parallel	42 [20, 22]	Perimenopausal women	45.2 (45.3, 45.1)	Capsule of a standardized fenugreek seed extract	6 weeks	0.5
Thomas, 2020	India	RCT, Parallel	41 [19, 22]	Postmenopausal women	53.96 (53.85, 54.1)	Capsule of Fenugreek seed powder	6 weeks	0.5
Pickering, 2022	Australia	RCT, Parallel	43 [20, 23]	Pre‐diabetic	58.89 (58.2, 59.7)	Film‐coated tablet of T. foenum‐graecum L. seed	12 weeks	0.5
Singh, 2022	India	RCT, Parallel	208 (113, 95)	Pre‐menopausal women with PCOS	18‐45 years	Furocyst® capsule (Fenugreek Seed Extract)	12 weeks	1
Hota, 2023	India	RCT, Parallel	204	T2DM	52.62 (53.03, 52.22)	Fenfuro® capsules	12 weeks	1
Rao, 2023	Brisbane and Gold Coast, Australia	RCT, Parallel	55 [26, 29]	Females aged between 25 and 45 years	34.26 (34.75, 33.73)	Libifem®, an extract of fenugreek	8 weeks	0.6
Gupta, 2024	India	RCT, Parallel	81 [39, 42]	T2DM	51.6 (50.69, 52.45)	Fenfuro® capsules	12 weeks	1
Thakurdesai, 2024	Australia	RCT, Parallel	153 [46, 47]	healthy, recreationally active male	30.75 (30.80, 30.70)	fenugreek seed extract	8 weeks	0.6

Abbreviations: NAFLD, non‐alcoholic fatty liver disease; RCT, randomized controlled trial; T2DM, type 2 diabtes mellitus.

### 3.3 Risk of Bias Assessment

Table 2 provides details of the risk assessment of eligible studies. Most of studies were at high risk of bias for at least one domain. Three studies were at the lowest risk of methodological bias according to Cochrane criteria [34, 48, 49]. All of the studies were at low‐risk for the sequence generation domain (supplementary Graph 1). Most studies were at high risk for conflict of interest. Subgroup analysis comparing high versus low risk‐of‐bias studies showed no statistically significant difference in effect estimates for any of the three outcomes.

**Table 2:** Study quality and risk of bias assessment.

First author, year	Risk of selection bias		Risk of performance bias	Risk of detection bias	Risk of attrition bias	Risk of reporting bias	Risk of other bias	
	Sequence generation	Allocation concealment	Blinding of performance	Blinding of outcome assessment	Incomplete outcome data	Selective reporting	Chance bias (baseline imbalance)	Conflict of interest
Babaei, 2020	**L**	**U**	**L**	**L**	**L**	**L**	**L**	**L**
Deshpande, 2020	**L**	**L**	**L**	**L**	**L**	**L**	**L**	**H**
Gupta, 2024	**L**	**L**	**L**	**L**	**L**	**L**	**H**	**H**
Hadi, 2020	**L**	**H**	**H**	**L**	**L**	**L**	**L**	**L**
Hota, 2023	**L**	**L**	**L**	**L**	**H**	**L**	**U**	**L**
Inanmdar, 2016	**L**	**L**	**L**	**L**	**L**	**L**	**L**	**L**
Lu, 2008	**L**	**L**	**L**	**L**	**H**	**H**	**L**	**L**
Pickering, 2022	**L**	**L**	**L**	**L**	**L**	**L**	**H**	**L**
Rao, 2023	**L**	**U**	**L**	**L**	**L**	**L**	**L**	**H**
Rao, 2019	**L**	**L**	**L**	**L**	**L**	**L**	**L**	**L**
Thomas, 2020	**L**	**L**	**L**	**L**	**L**	**L**	**L**	**H**
Thakurdesai, 2024	**L**	**U**	**L**	**L**	**L**	**L**	**L**	**H**
Wankhede, 2016	**L**	**U**	**L**	**L**	**L**	**L**	**L**	**H**
Verma, 2016	**L**	**L**	**L**	**L**	**H**	**H**	**L**	**H**
Khanna, 2020	**L**	**L**	**L**	**L**	**L**	**L**	**L**	**H**
Singh, 2022	**L**	**L**	**L**	**L**	**L**	**H**	**L**	**H**

Abbreviations: H, high risk; L, low risk; U, unclear.

The overall certainty of evidence for all outcomes (ALP, AST, and ALT) was rated as very low (Table 3). This was mainly due to serious risk of bias, as a substantial proportion of included studies were judged to be at high or unclear risk in at least one domain. Additional downgrading was applied for indirectness (with most participants originating from a limited number of countries) and imprecision (confidence intervals crossing the null effect).

**Table 3:** Certainty assessment.

Certainty assessment						Certainty
№ of studies	Risk of bias	Inconsistency	Indirectness	Imprecision	Publication bias	
**ALP**						
14	Serious^a^	Not serious^b^	serious^c^	Serious^d^	Not serious	Very Low
**AST**						
16	Serious^e^	Not serious^f^	serious^g^	Serious^h^	Not serious	Very Low
**ALT**						
14	Serious^i^	Not serious^j^	Serious^g^	Serious^h^	Not serious	Very Low

*Note:* ^a^Nearly 77% of studies and participants were at high risk of bias, therefore it was downgraded one level. ^b^Although *I* ^2^ was 70.7%, we did not downgrade for inconsistency since in the subgroup with a dose < 1 g/d (SMD: 0.03 (95% CI: −0.45 to 0.03, *I* ^2^: 11.5) with seven studies heterogeneity was reduced. ^c^Downgraded one level, since > 50% of participants were from India. ^d^The effect size for fenugreek on ALP levels (SMD: −0.21, 95% CI: −0.44 to 0.03) cross the null effect, so downgraded one level. ^e^Nearly 81% of studies and participants were at high risk of bias, therefore it was downgraded one level. ^f^ *I* ^2^ was less than 50% (33%). ^g^Downgraded one level, since > 50% of participants were from India. ^h^The confidence intervals (−0.12 to 0.18) cross the null effect, so downgraded one level. ^i^Nearly 78.5% of studies and participants were at high risk of bias, therefore it was downgraded one level. ^j^ *I* ^2^ was less than 50% (33%).

### 3.4 Effect of Fenugreek Supplementation on ALP

Fourteen studies with 1102 participant were included in the analysis. No significant reduction was observed in ALP (SMD = −0.21, 95% CI = −0.44 to 0.03) with the heterogeneity of 70.7%. Sensitivity analyses confirmed that the results were robust across different studies. There was no heterogeneity between subgroups, except for a dose. A significant greater reduction was observed for a dose of more than 1 g/day. Results of subgroup analysis have been presented in Table 4. No publication bias was detected by funnel plot (Figure S1) and Egger's test (*p* = 0.33).

**Table 4:** Subgroup analysis of studies investigated the effect of Fenugreek on liver enzymes.

Subgroup factor		Num of studies	MD*, 95% CI	*I2*%, *p* heterogeneity	*p* between group
**ALP**					
All studies		14	−0.21 (−0.44 to 0.03)	11.6	
Duration	≥ 12 wks.	9	−0.34 (0.63 to −0.05)	75.2, < 0.001	0.08
	< 12 wks.	5	−0.06 (−0.29 to 0.42)	40.5, 0.15	
Age	≥ 30 years	9	−0.14 (−0.40 to 0.13)	57.3, 0.02	
	< 30 years	3	−0.11 (−0.35 to 0.14)	76.	
Dose	≥ 1 g/d	7	−0.44 (−0.78 to −0.10)	78.6, < 0.001	0.03
	< 1 g/d	7	0.03 (−0.45 to 0.03)	11.5, 0.34	
Health conditions	Metabolic disorders	7	−0.36 (0.71 to −0.02)	79.8, < 0.001	0.15
	No metabolic disorders	7	−0.04 (−0.31 to 0.24)	35.9, 0.16	
BMI	Overweight/obese	9	−0.11 (0.45 to 0.22)	77.7, < 0.001	0.62
	Normal weight	5	−0.23 (0.51 to 0.06)	0.0, 0.73	
Baseline levels	< 129	8	−0.12 (−0.36, 0.13)	56.9, 0.02	0.43
	≥ 129	6	−0.32 (−0.78, 0.13)	75.5, 0.001	
Risk of bias	Low	3	−0.11 (−0.53, 0.30)	31.1, 0.23	0.25
	High	11	−0.23 (−0.51, 0.05)	75.4, < 0.001	
**AST**					
All studies		16	0.03 (−0.12 to 0.18)	33.2, 0.10	
Duration	≥ 12 wks.	10	−0.18 (−2.22, 1.86)	51.2, 0.03	0.28
	< 12 wks.	6	0.17 (−0.10 to 0.44)	0.0, 0.53	
Age	≥ 30 years	11	0.09 (−0.07 to 0.24)	0.0, 0.67	0.85
	< 30 years	3	0.12 (−0.24 to 0.49)	0.0, 0.45	
Dose	≥ 1 g/d	7	−0.08 (−0.30 to 0.15)	53.5, 0.04	0.09
	< 1 g/d	9	0.18 (−0.02 to 0.39)	0.0, 0.79	
Health conditions	Metabolic disorders	8	−0.49 (−3.04 to 2.07)	59.1, 0.02	0.21
	No metabolic disorders	8	0.16 (−0.05 to 0.37)	0.0, 0.76	
BMI	Overweight/obese	9	0.06 (−2.32 to 2.45)	58.4, 0.01	0.61
	Normal weight	7	0.09(−0.10 to 0.29)	0.0, 0.62	
Risk of bias	Low	3	0.19, (−0.13, 0.51)	0.0, 0.79	0.33
	High	13	0.01 (−0.17, 0.18)	41.2, 0.06	
**ALT**					
All studies		15	−1.41 (−3.07, 0.26)	24.9, 0.18	
Duration	≥ 12 wks.	9	−1.12 (−3.55 to 1.31)	25.4, 0.22	0.80
	< 12 wks.	6	−1.57 (−4.08 to 0.95)	34.9, 0.17	
Age	≥ 30 years	11	−1.92(−3.81 to −0.04)	17.8, 0.27	0.92
	< 30 years	3	−1.75 (−4.55 to 1.05)	2.8, 0.36	
Dose	≥ 1 g/d	6	0.37 (−3.07 to 3.81)	24.7, 0.25	0.18
	< 1 g/d	9	−2.26 (−3.97 to −0.55)	12.7, 0.33	
Health conditions	Metabolic disorders	7	0.49 (−2.47 to 3.44)	10.9, 0.35	0.11
	No metabolic disorders	8	−2.36 (−4.13 to −0.59)	16.4, 0.30	
BMI	Overweight/obese	8	0.11 (−2.12 to 2.33)	0.0, 0.58	0.02
	Normal weight	6	−3.44 (−5.21 to −1.66)	0.0, 0.75	
Risk of bias	Low	3	−3.32 (−6.15, −0.49)	0.0, 0.42	0.16
	High	12	−0.85 (−2.81, 1.10)	28.5, 0.17	

Abbreviations: ALP, alkaline phosphates; ALT, alanine transferase; AST, aspartate transferase.

* Standardized mean difference for ALP and weighted mean difference for AST and ALT.

### 3.5 Effect of Fenugreek Supplementation on AST

Analysis of 16 RCTs with 1324 participants revealed no significant change in serum AST (WMD = 0.03 IU/L, 95% CI = − 0.12 to 0.18) with the heterogeneity of 33.2%. Sensitivity analyses were performed to assess the robustness of the findings, which confirmed that the overall effect remained non‐significant even when excluding outlier studies. There was no heterogeneity between subgroups. The baseline levels of AST in all included studies were within the normal standard range, therefore, conducting subgroup analysis by the baseline values was not possible (Table 4). No publication bias was detected through inspection of funnel plot (Supplementary Fig. 2) and Egger's test (*p* = 0.16).

### 3.6 Effect of Fenugreek Supplementation on ALT

Analysis of fourteen studies with 963 participants indicated no significant change in serum ALT (WMD = −1.41 IU/L, 95% CI −3.07 to 0.26) with a heterogeneity of 24.9%. The summary estimate remained unchanged after sequential omission of each study from the main analysis. Subgroup analyses revealed no significant heterogeneity of between groups, except for BMI, where a greater reduction in ALT was observed among studies with participants of normal BMI. The baseline levels of AST in all included studies except one did not exceed the maximum threshold (Table 4). The funnel plot (Figure S3) and Egger's test for asymmetry (*p* = 0.02) suggested potential small‐study effects. However, in trim‐and‐fill analysis no study was included. This discrepancy may reflect the higher sensitivity of Egger's test and the limited power of trim‐and‐fill. Therefore, the possibility of publication bias cannot be excluded, and results should be interpreted cautiously.

## 4 Discussion

The current study was conducted as a systematic review of the 16 published randomized controlled trials concerning the impacts of fenugreek (Trigonella foenum‐graecum) on some liver function tests like AST, ALT, and ALP. The results have indicated that fenugreek does not make statistical changes in the serum levels of AST and ALT. The observed changes in ALT (− 1.41 IU/L) and AST (0.03 IU/L) are small and unlikely to be clinically meaningful, particularly in populations with normal baseline levels. For ALP, although no significant effect was observed overall, the subgroup receiving ≥ 1 g/day showed a small‐to‐moderate reduction (SMD ≈ −0.44). However, because this estimate is based on the standardized mean differences and is accompanied by substantial heterogeneity, its clinical relevance remains uncertain.

Substantial heterogeneity was observed for ALP. However, the direction of effect was consistent across most studies (all but one showed a reduction or no change in ALP). Subgroup analyses exploring baseline ALP levels, health status, BMI, age, and fenugreek formulation did not identify significant sources of heterogeneity. Therefore, the residual heterogeneity remains unexplained and may be due to unmeasured factors or random variation. Accordingly, findings from the high‐dose subgroup (I^2^ = 78.6%) should be interpreted with caution.

There are several preclinical and clinical studies assessing fenugreek's hepatoprotective action in different conditions and diseases [30, 47, 48, 50, 51, 52, 53, 54]. Fenugreek is recognized for its rich array of phytochemicals that contribute to its potential hepatoprotective effects. It contains various bioactive compounds, including saponins, flavonoids, phenolic acids, and the key amino acid 4‐hydroxyisoleucine. These constituents play significant roles in modulating liver enzymes and improving liver function.

For example, diosgenin has been shown to inhibit the lipid accumulation in the liver by means of liver‐X‐receptor‐alpha antagonism. This action decreases the mRNA expression of lipogenic genes and subsequently ameliorates hepatic steatosis and hyperlipidemia. Moreover, the antioxidant action of some flavonoids and phenolic acids may also aid in the reducing oxidative stress in the liver thereby protecting the liver from damage. The synergistic effect of the constituents increases fenugreek's ability to control lipid metabolism, reduce blood lipids, increase sensitivity to insulin, and therefore be a potent natural adjuvant in the treatment of liver disorders [10, 55, 56, 57, 58]. Additionally, a recent systematic review found that polyphenol supplementation can both prevent and ameliorate NAFLD [59].

An increase in ALT and AST reflects hepatocellular injury, and in populations with only mild or subclinical liver dysfunction fenugreek may not produce measurable changes in these enzymes. Several factors can explain the discrepancy between preclinical hepatoprotective findings and the null enzyme results in clinical studies. Study heterogeneity (differences in design, duration, fenugreek preparation and dose), variation in participant characteristics (age, comorbidities, baseline liver function), and inadequate control of confounders (diet, alcohol intake, physical activity, concomitant medications) can all mask modest biochemical effects [60]. Short treatment durations and insufficient dosing in human trials may be particularly important: animal studies often use higher relative doses and longer exposures than feasible in clinical settings [61, 62]. Finally, ALT and AST are crude markers of liver health and may not capture improvements in oxidative stress, inflammation, or histology; thus, fenugreek could exert hepatoprotective effects not reflected by serum transaminases [44, 63, 64].

Our subgroup analyses indicate that fenugreek has a positive impact on biliary health and enzymatic activity when the dosage is exceeded 1 g per day. This observation reinforces the concept of functional food with therapeutic application of fenugreek in cases of elevated ALP. Still, the exact reasons behind this remain to be determined. ALP is an enzyme located on several tissues including the liver, bile duct, and bone, where it is heavily concentrated. Its fundamental role is carrying out the dephosphorylation of a range of substances, and therefore collaboration in bile production and excretion, and various substrates in the liver that undergo metabolism. Augmented ALP levels may serve as a marker for cholestasis [65, 66, 67]. Hence, the fenugreek supplementation effects on declining ALP level may indicate enhanced hepatic function through either increased bile production or improved bile outflow within the liver.

As stated earlier, the systematic review and meta‐analysis of fenugreek's impact on liver function tests provided some insights while balancing the study's fenugreek dosage and duration of intervention highlights its limitations. The included clinical trials were heterogeneous in terms of dosage, intervention duration, and other demographic and clinical characteristics of the patients which could impact study outcome measures. In addition, a main proportion of the studies did not monitor or report participants’ lifestyle modification, including any change in physical activity and dietary pattern (mainly consumed fat and carbohydrate, and daily calorie intake). This can be an important confounding factor. Moreover, the quality of the individual trials varied, with some exhibiting a high or unclear risk of bias in different domains that may affect the reliability of their findings. Particularly, most of the trials had the risk of detection bias (i.e., having no perfect blinding of outcome assessment). Additionally, incomplete outcome data (known as risk of attrition bias) and selective reporting (i.e., risk of reporting bias) were detected in about one‐fourth of the included studies. No measurement of other objective hepatic outcome measures in the majority of assessed studies should be considered as an important limitation. In detail, assessment of steatosis percent, steatosis stage, fibro score, and controlled attenuation parameter (CAP) score using elastography by fibroscan and measuring serum albumin level were not performed in most studies. The mentioned limitations highlight the need for more rigorous clinical trials with standardized protocols and measuring different objective measures to validate our findings.

### 4.1 Strengths and Limitations

Our review has several strengths: a comprehensive, up‐to‐date search of three major databases, by pooling 16 trials totaling 1171 participants, minimized the likelihood of missing relevant trials, and inclusion of only controlled clinical trials increased the clinical relevance of the findings. Additionally, focusing on standardized, routinely measured liver enzymes (ALP, ALT, AST) enhanced comparability across studies and clinical interpretability, and subgroup analysis revealing an ALP reduction at doses > 1 g/day offers a useful hypothesis about dose‐dependent effects.

As stated earlier, the systematic review and meta‐analysis of fenugreek's impact on liver function tests provided some insights while balancing the study's fenugreek dosage and duration of intervention highlights its limitations. The included clinical trials were heterogeneous in terms of several demographic and clinical characteristics of the patients. In addition, a main proportion of the studies did not monitor or report participants’ lifestyle modification, including any change in physical activity and dietary pattern (mainly consumed fat and carbohydrate, and daily calorie intake). This can be an important confounding factor. Most included studies had high or unclear risk of bias in at least one domain, particularly regarding allocation concealment and potential conflicts of interest, which reduces confidence in the findings. Accordingly, the certainty of the evidence was rated as very low using the GRADE approach, primarily due to serious risk of bias, as well as concerns regarding indirectness and imprecision. Therefore, our findings should be interpreted with caution, and further high‐quality randomized controlled trials are required to provide more definitive evidence. No measurement of other objective hepatic outcome measures in the majority of assessed studies should be considered as an important limitation. In detail, assessment of steatosis percent, steatosis stage, fibro score, and controlled attenuation parameter (CAP) score using elastography by fibroscan and measuring serum albumin level were not performed in most studies.

### 4.2 Future Perspectives

To clarify fenugreek's potential as a hepatoprotective medicinal plant and address limitations identified in existing trials, future research should target the following areas:

– Trials in populations with elevated liver enzymes: It is essential to run randomized controlled trials specifically enrolling patients with biochemical or imaging evidence of liver injury (e.g., elevated ALT/AST, confirmed NAFLD/NASH) to determine whether fenugreek produces clinically meaningful improvements in individuals who stand to benefit most.
– Standardized dosing regimens: Use of standardized, well‐characterized fenugreek preparations and dosing schedules is recommended. Trials should report extract composition (e.g., diosgenin, polyphenol content), dose in mg/kg and absolute dose, and justify dose selection using pharmacokinetic or preclinical data to permit dose–response assessment and cross‐study comparison.
– Longer intervention duration: Future research should implement longer treatment periods and follow‐up (months rather than weeks) to capture slow‐developing improvements in liver histology, steatosis, and metabolic parameters that short‐term studies may miss.
– Use of objective liver outcome measures: Researchers should move beyond serum transaminases as the sole endpoints. They should incorporate noninvasive quantitative assessments—transient elastography (FibroScan) with liver stiffness and controlled attenuation parameter (CAP) scores, Magnetic Resonance Imaging Proton Density Fat Fraction (MRI‐PDFF), and, where ethically feasible, histological evaluation—to detect changes in fibrosis, steatosis, and inflammation that ALT/AST may not reflect.
– Safety monitoring at higher or prolonged doses: As future trials explore higher doses or longer exposures, they should include predefined safety monitoring protocols (serial liver biochemistry, renal function, adverse event reporting, and, if indicated, drug–nutrient interaction assessments). Robust safety data will be essential to define tolerability and guide clinical use.

Collectively, these design improvements will strengthen causal inference, enable mechanistic insights, and determine the clinical relevance of fenugreek supplementation for liver disease management.

## 5 Conclusion

In conclusion, our findings indicate that fenugreek does not significantly affect traditional liver enzyme markers under current dosing regimens for most studied populations. However, there is potential benefit at higher doses regarding specific enzymatic activities related to biliary functions rather than solely hepatocellular integrity. However, given the low certainty of evidence and the high risk of bias in several domains across most included studies, these conclusions should be interpreted cautiously until confirmed by more rigorous trials. Further research should be performed to elucidate fenugreek's phytochemicals for its hepatoprotective properties. Additionally, exploring its effective doses for liver disorders in more homogeneous populations along with considerations about its safety could provide valuable insights into its therapeutic potential.

## Author Contributions

**Mohsen Mohit:** conceptualization, investigation, methodology, writing – review and editing. **Melika Mahmoudizadeh:** methodology, investigation, writing – original draft. **Mohammad Hashem Hashempur:** conceptualization, methodology, writing – review and editing. **Mitra Soltani:** methodology, writing – original draft. **Asma Kazemi:** investigation, methodology, writing – review and editing, formal analysis.

## Funding

The authors have nothing to report.

## Conflicts of Interest

The authors declare no conflicts of interest.

## 1 Transparency Statement

Mohsen Mohit affirms that this manuscript is an honest, accurate, and transparent account of the study being reported; that no important aspects of the study have been omitted; and that any discrepancies from the study as planned have been explained.

## Supporting information


**Figure S1:** Funnel plots showing study precision against standardized mean difference and 95% confidence intervals for the effect of fenugreek on alkalin phosphatase.

**Figure S2:** Funnel plots showing study precision against standardized mean difference and 95% confidence intervals for the effect of fenugreek on aspartate aminotransferase.

**Figure S3:** Funnel plots showing study precision against standardized mean difference and 95% confidence intervals for the effect of fenugreek on alanine aminotransferase.

**Supplementary Graph 1:** Summary of methodological quality and risk of bias assessment for included studies.

## Data Availability

The datasets analyzed in this study are available from the corresponding author upon reasonable request.
