# Supplementary material for: The Effect of Fenugreek (Trigonella foenum‐graecum) Supplementation on Liver Function Enzymes; a Systematic Review and Meta‐Analysis of Controlled Clinical Trials
Source: Health Sci Rep. 2026 Aug 4;9(8):e72960. doi: 10.1002/hsr2.72960 (PMC13438187; doi:10.1002/hsr2.72960)
Supplement: Supplementary file 1 [file HSR2-9-e72960-s003.docx]

**Gragh 1.** Study quality and risk of bias assessment

Risk of bias graph

|  | Low risk of bias | Unclear risk of bias | High risk of bias |
| --- | --- | --- | --- |
| Risk of conflict of interest | 7 | 0 | 9 |
| Risk of Chance bias (baseline imbalance) | 13 | 1 | 2 |
| Risk of reporting bias (Selective reporting) | 13 | 0 | 3 |
| Risk of attrition bias (Incomplete outcome data) | 13 | 0 | 3 |
| Risk of detection bias (Blinding of outcome assessment) | 3 | 0 | 13 |
| Risk of performance bias (Blinding of performance) | 15 | 0 | 1 |
| Allocation concealment | 11 | 4 | 1 |
| Sequence generation | 16 | 0 | 0 |
